# Inhibiting biofilm formation by *Klebsiella pneumoniae* B5055 using an iron antagonizing molecule and a bacteriophage

**DOI:** 10.1186/1471-2180-13-174

**Published:** 2013-07-26

**Authors:** Sanjay Chhibber, Deepika Nag, Shruti Bansal

**Affiliations:** 1 Department of Microbiology, Basic Medical Sciences (BMS) Block, Panjab University, 160014 Chandigarh, India

**Keywords:** Resistance, Treatment, Phage depolymerase, Adjunct, Alternate therapy

## Abstract

**Background:**

Success of biofilm dwelling bacteria in causing persistent and chronic infections is attributed to their resistance towards antibiotics and immune defences. Free iron is critical for the growth of biofilm associated bacteria. Therefore in the present study, the effect of limiting iron levels by addition of divalent Co[II] ions in combination with a bacteriophage was used for preventing/disrupting *Klebsiella pneumoniae* biofilms.

**Results:**

A significantly higher reduction (p < 0.005) in bacterial numbers in the younger as well as older biofilms treated with Co[II] and depolymerase producing phage in combination was observed in comparison to when either of the agents was used alone. The role of phage borne depolymerase was confirmed, as an insignificant eradication of biofilm by non-depolymerase producing bacteriophage in combination with cobalt ions was observed. The results of viable count were further confirmed by visual examination of biofilms.

**Conclusion:**

From the study it can be concluded, that iron antagonizing molecules and bacteriophages can be used as adjunct therapy for preventing biofilm development.

## Background

Biofilms are cell-cell or solid surface-attached assemblages of microbes that are entrenched in a hydrated, self-produced matrix [1]. Bacteria growing in biofilms exhibit increased resistance to antimicrobials and host immune response compared to their freeliving, planktonic counterparts due to several reasons like restricted penetration of antimicrobials into a biofilm, decreased growth rate, and expression of possible resistance genes [2]. *Klebsiella pneumoniae* is an important biofilm forming organism responsible for a wide range of infections placing it among the eight most important nosocomial pathogens [3]. The threat of antibiotic resistance and their inability in breaking the biofilm structure has increased the likelihood that novel strategies for preventing or delaying the biofilm growth mode are urgently needed [4].

Bacteriophages infect bacteria, hijack their machinery, replicate intracellularly and are released by host cell lysis. They offer various advantages over antibiotics as antibiofilm agents because of their specific, non-toxic, self replicating and self limiting nature [5,6]. Phage borne depolymerases degrade biofilm exopolysaccharide matrix that acts as a barrier for antimicrobials, infect the organisms and cause extensive biofilm disruption [7]. Since phages are rapidly removed from circulation once injected/ingested, are unable to penetrate the older biofilms which contain large number of metabolically inactive cells [8] thus it can be said that either phages or antibiotics when used alone do not stand a chance especially against biofilm associated bacterial infections. Therefore, treating biofilms with combinations of chemically distinct antimicrobials might be an effective strategy to kill some of these different cell types.

Iron is an essential factor in bacterial growth participating in oxygen and electron transport processes, essential for biofilm formation in bacteria [9,10] where it regulates surface motility, promotes biofilm formation by stabilizing the polysaccharide matrix [11] and is considered critical for transition from planktonic to sessile existence. Thus, reducing iron availability has been proposed as a potential means to impair biofilm development by *K*. *pneumoniae*, *Pseudomonas aeruginosa*, *Escherichia coli* etc. [12-15]. In light of this emerging perspective, we undertook the present study to explore the possibility of using an iron antagonizing molecule and a bacteriophage alone as well as in combination to inhibit biofilm formation by *K*. *pneumoniae* B5055.

## Methods

### Bacterial strain, phages and growth conditions

*K*. *pneumoniae* B5055 (O1:K2) obtained originally from Dr. Mathia Trautmann, Department of Medical Microbiology and Hygiene, University of Ulm, Germany; KPO1K2 and NDP, depolymerase and non-depolymerase producing phages against *K*. *pneumoniae* B5055, previously characterized in our laboratory [16-18] were used in the present study. As reported earlier by Verma et al. [16] phage KPO1K2 possesses icosahedral head with pentagonal nature with apex to apex head diameter of about 39 nm. It has a genome of 42 kbps, a short noncontractile tail (10 nm) and a T7 like structural protein pattern suggesting its inclusion into family *Podoviridae* with a designation of T7-like lytic bacteriophage.

The titre of the bacteriophage preparation was estimated by the soft agar overlay method [19] and was expressed as plaque forming units/ml (pfu/ml). Nutrient broth was used routinely for bacterial culture; bacterial dilutions were made in sterile 0.85% sodium chloride (NaCl) whereas dilutions of phage were made in sterile Phosphate Buffer Saline (PBS). Biofilm experiments in 96 well microtiter plates as well as on cover slips were conducted in M9 minimal medium [composition/100 ml: disodium hydrogen phosphate (Na_2_HPO_4_)–65 mg, potassium dihydrogen phosphate (KH_2_PO_4_)–150 mg, sodium chloride (NaCl)-25 mg, ammonium chloride (NH_4_Cl)–50 mg, magnesium sulphate (MgSO_4_)–12 mg, calcium chloride (CaCl_2_)–0.5 mg, glucose −200 mg] and iron (FeCl_3_) was supplemented as indicated. The divalent metal ion containing salt, CoSO_4_ was used as the iron antagonizing molecule at a concentration of 500 μM.

### Biofilm growth on microtiter plates

*K*. *pneumoniae* biofilms were grown in 96-well microtiter plate according to method described by Bedi et al. [20]. Briefly, 100 μl of minimal M9 medium and 100 μl of bacterial culture (OD_600_ = 0.3) equivalent to 10^8^ CFU/ml of *K*. *pneumoniae* were added to the wells of microtiter plate and incubated at 37°C overnight. In each test, control wells containing sterile minimal media were included that acted as plate sterility control. After every 24 h, planktonic bacteria were removed and a set of two wells (corresponding to each day) were washed thoroughly 3 times with 0.85% NaCl. Adherent biofilms were scraped from 2 wells, suspended in 0.85% NaCl and vortexed for 3 min using Remi Cyclomixer (Remi Instruments & Appliances Ltd, Bombay, India). Microbial load of biofilm was enumerated by viable cell counting. In rest of the wells, spent medium was replaced with fresh sterile M9 media and plate was reincubated at 37°C overnight. This procedure was repeated until 7^th^ day of experiment.

### Biofilm growth in iron supplemented minimal media

Different wells of 96-well microtiter plate were inoculated with 100 μl of *K*. *pneumoniae* culture (OD_600_ = 0.3) equivalent to a bacterial cell density of 10^8^ CFU/ml and 100 μl of M9 media supplemented with different concentrations of FeCl_3_ (0, 10 μM, 100 μM, 1000 μM). After overnight incubation at 37°C contents of all wells were removed and from two set of wells containing 0/10 μM/100 μM/1000 μM FeCl_3_ supplemented minimal media unadhered bacteria were washed off, biofilms were scraped from 8 wells, cells were enumerated by plating on nutrient agar plates. In rest of the wells, spent medium was replaced with fresh sterile M9 media and plate was reincubated at 37°C overnight. This procedure was repeated until 7^th^ day of experiment.

### Biofilm growth in iron supplemented minimal media with cobalt addition

To determine the efficacy of Cobalt sulphate (CoSO_4_) in inhibiting the biofilm growth, 100 μl of *K*. *pneumoniae* was inoculated in different wells of microtiter plate containing 100 μl of minimal media supplemented with 10 μM FeCl_3_ or 500 μM of Cobalt sulphate (CoSO_4_) alone or in combination. After overnight incubation at 37°C contents of all wells were removed and from two set of control wells and wells with 10 μM FeCl_3_/500 μM CoSO_4_/both, supplemented minimal media (8 samples) unadhered bacteria were removed and viable counts were determined. Reduction in log values of bacterial count was noted in comparison to untreated control. In rest of the wells, spent medium was replaced with fresh media and plate was reincubated at 37°C overnight. This procedure was repeated until 7^th^ day of experiment.

### Bacteriophage treatment of biofilm grown in minimal media supplemented with cobalt (CoSO_4_) and iron (FeCl_3_) salts

To determine the efficacy of bacteriophage alone as well as in combination with the iron anatagonizing molecule in treating the biofilms of *K*. *pneumoniae* B5055, 100 μl of bacterial culture was inoculated in different wells of microtiter plate containing 100 μl of minimal media supplemented with 10 μM FeCl_3_ and/or 500 μM of Cobalt sulphate (CoSO_4_) and incubated at 37°C overnight. Unadhered bacteria were removed from two set of wells supplemented with 10 μM FeCl_3_ and 10 μM FeCl_3_+ 500 μM CoSO_4_ on different days. Thereafter, these biofilms were exposed to bacteriophage (KPO1K2/NDP) at multiplicity of infection [m.o.i: ratio of infectious agent (e.g. phage or virus) to infection target (e.g. bacterial cell)] of 1 for 3 h followed by washing with 0.85% NaCl and enumeration of viable cells from 8 wells. A set of two wells containing biofilm grown in unsupplemented, iron supplemented minimal media alone and with the addition of CoSO_4_ served as controls and were also processed as mentioned previously on each day. In rest of the wells, spent medium was replaced with fresh media and plate was re-incubated at 37°C overnight. This procedure was repeated until 7^th^ day of experiment.

### Development of biofilm on glass coverslip

To determine the effectivness of treatment with various combinations qualitatively, biofilms were grown on glass coverslips (18 mm × 18 mm; 0.08–0.12 mm; Corning Glass, USA) at air–liquid interface by the Tipbox batch culture method of Hughes et al. [7] as standardized in our laboratory by Verma et al. [18]. Tip-box mounted coverslips and minimal M9 media supplemented with 10 μM FeCl_3_ with or without 500 μM CoSO_4_ were sterilized separately. 100 μl bacterial culture (10^8^ CFU/ ml) was added to the media which was then poured into the tip box. The whole set-up was incubated at 37°C. Spent growth medium in the culture boxes was replaced every 24 h. On 3^rd^ and 7^th^ day 16 coverslips (4 corresponding to each group) were removed, rinsed thoroughly with sterile 0.85% NaCl and 8 were incubated with bacteriophage (MOI = 1) for 3 hours. After treatment, biofilm laden coverslip was washed with sterile sodium phosphate buffer (pH 7.2), stained for 15 min in dark with the components of LIVE/DEAD BacLight Bacterial Viability Kit (Invitrogen), washed with 0.85% NaCl and observed under oil immersion 100× objective, with a B2A filter set fitted in a fluorescent microscope (Nikon). The images were captured using an image acquisition system by Nikon. The untreated cover-slips were also processed in a similar way as treated ones. 8 cover slips (2 for each group) were processed for ascertaining the viable cell count of the treated as well as untreated biofilm.

### Statistical analysis

All experiments were performed in duplicate and repeated at least three times on different days. The bacterial count was log_10_ transformed as described by Anderl et al. [21]. On different days of biofilm formation, all the data from a particular treatment and from particular time points were grouped separately and the log reductions in comparison to untreated biofilm at the respective time points were calculated. The effect of different treatments on biofilm eradication was evaluated by the Student’s t-test and P < 0.05 was considered significant. Data were analyzed using Excel software.

## Results

### Establishment of biofilms on microtiter plates in iron supplemented media

**Figure 1:**
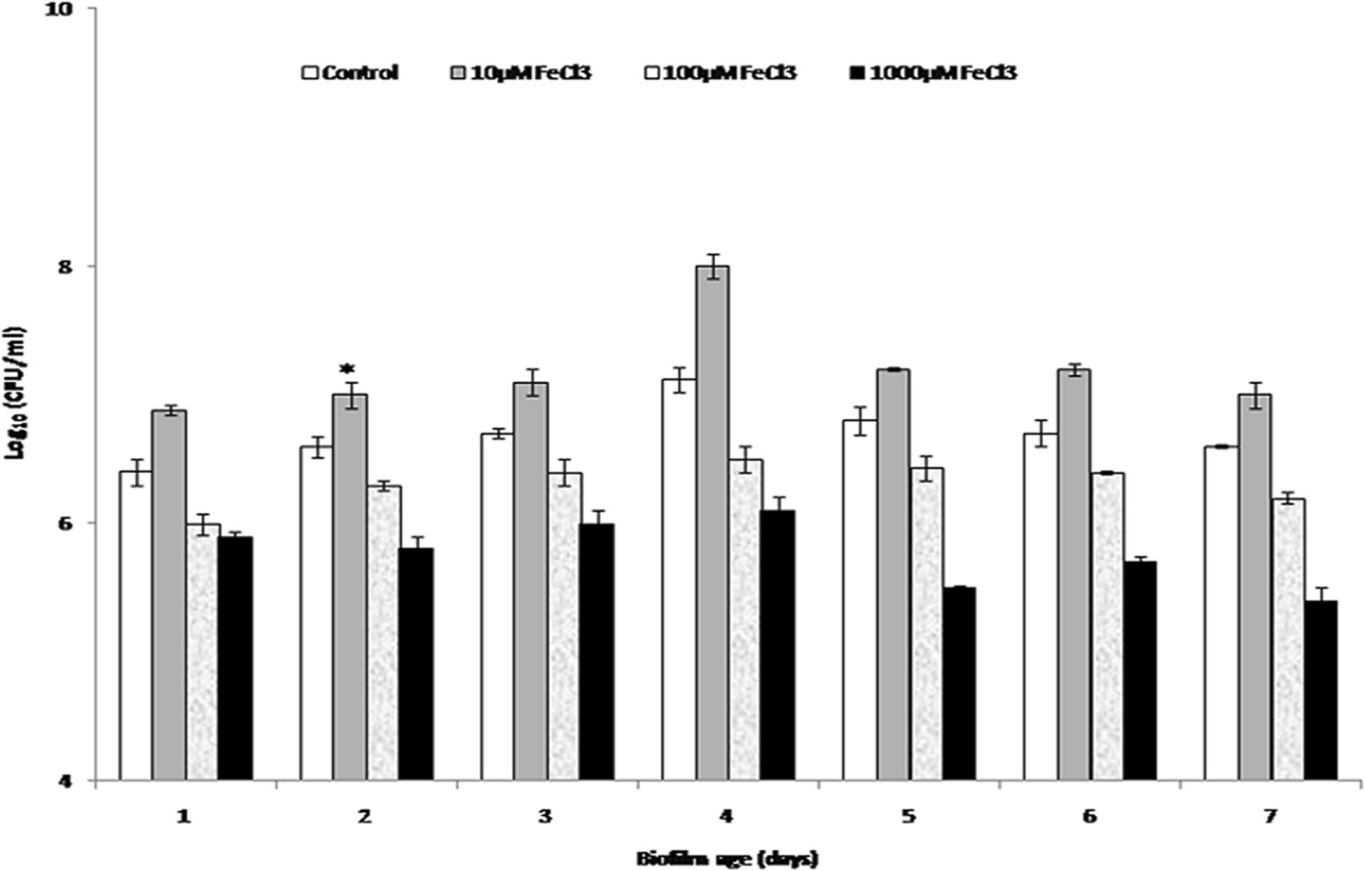
**Kinetics of biofilm formation by *K. pneumoniae *B5055 grown in minimal media (M9) with or without supplementation of FeCl**_**3**_**.** *p < 0.05 (10 μM FeCl_3_ vs control group).

*K*. *pneumoniae* biofilms was established in minimal (M9) media and bacterial count was enumerated on various days. Initially, bacterial count for the young immature 2^nd^ day biofilms was 6.7 ± 0.08 Log_10_ CFU/ml followed by a peak on day 4 (7.12 ± 0.04 Log_10_ CFU/ml) and a further decline resulting in a bacterial count of 6.6 ± 0.10 Log_10_ CFU/ml on 7^th^ day for the older mature biofilm. The effect of supplementation with different concentrations of FeCl_3_ in minimal media was studied on the biofilm growth. Addition of 10 μM FeCl_3_ enhanced the growth as a significant increase (p < 0.05) in the bacterial count was observed 2^nd^ day onwards (Figure 1) in comparison with non-iron supplemented control wells. This increase was consistent throughout the incubation period. On the contrary, wells supplemented with 100 and 1000 μM of FeCl_3_ showed reduction 4^th^ day onwards with respect to control and 10 μM FeCl_3_ containing wells.

### Antimicrobial treatment of biofilms grown on microtiter plates

**Figure 2:**
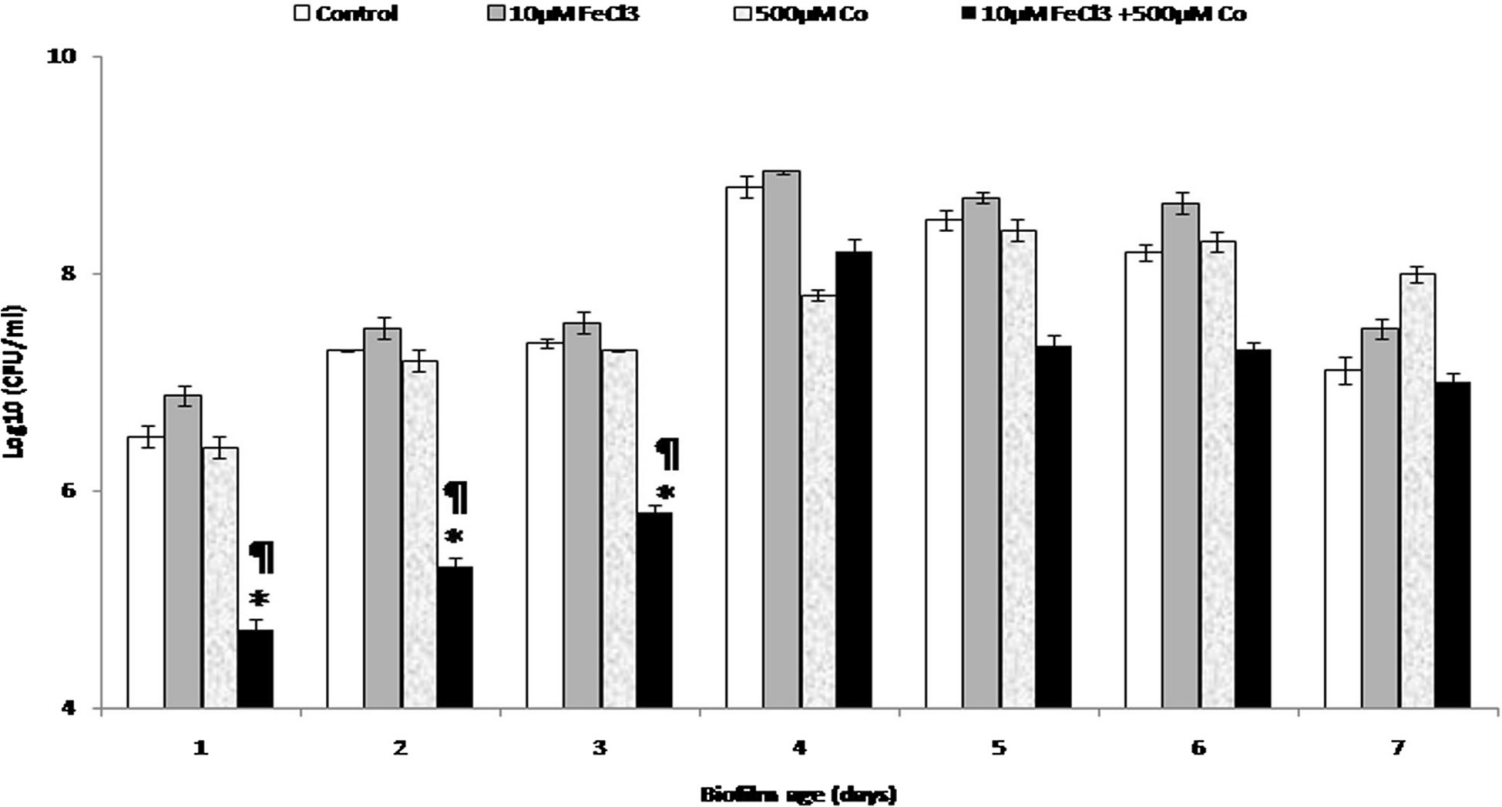
**Kinetics of biofilm formation by *K. pneumoniae *B5055 grown in minimal media (M9) containing cobalt salt (CoSO**_**4**_**) or FeCl**_**3 **_**alone and in combination.** *p < 0.05 (10 μM FeCl_3_ + 500 μM CoSO_4_ vs 10 μM FeCl_3_), ^¶^*p* < 0.05 (10 μM FeCl_3_ + 500 μM CoSO_4_ vs 500 μM CoSO_4_).

The effect of addition of iron antagonizing molecule i.e. CoSO_4_ on *K*. *pneumoniae* B5055 biofilms grown in minimal media supplemented with 10 μM FeCl_3_ was studied and it was observed that although supplementation with 10 μM FeCl_3_ resulted in significant enhancement of biofilm growth but addition of 500 μM chelator alone exerted minimal inhibitory effect on biofilm growth in comparison to control wells containing no iron or chelator. When a combination of 10 μM FeCl_3_ and 500 μM CoSO_4_ was added together, there was a significant decrease (p < 0.05) of ~2 logs in the younger biofilms till 3^rd^ day but the reduction decreased to ~ 1 log from 5^th^ day onwards for the older biofilms in comparison to the control wells containing no FeCl_3_ and CoSO_4_ (Figure 2).

**Figure 3:**
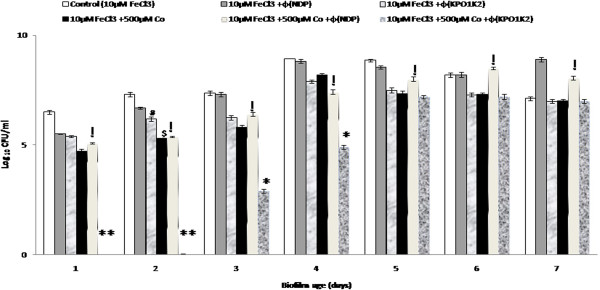
**Kinetics of biofilm formation by *K. pneumoniae *B5055 grown in minimal media (M9) supplemented with 10** **μM FeCl**_**3 **_**and treated with 500** **μM cobalt salt (CoSO**_**4**_**) and bacteriophage (KPO1K2)/ (NDP) alone as well as in combination.** *p < 0.05 [(10 μM FeCl_3_ +500 μM CoSO_4_ + Ø(KPO1K2) vs 10 μM FeCl_3_/10 μM FeCl_3_+ 500 μM CoSO_4_/10 μM FeCl_3_+ Ø(KPO1K2)], **p < 0.005 [(10 μM FeCl_3_ +500 μM CoSO_4_ + Ø(KPO1K2) vs 10 μM FeCl_3_/10 μM FeCl_3_+ 500 μM CoSO_4_/10 μM FeCl_3_+ Ø(KPO1K2)], ^#^*p* < *0*.*05* [(10 μM FeCl_3_ + Ø(KPO1K2) vs 10 μM FeCl_3_], ^$^*p* < *0*.*05*[(10 μM FeCl_3_ +500 μM CoSO_4_) vs 10 μM FeCl_3_], ^!^p > 0.05[(10 μM FeCl_3_ +500 μM CoSO_4_ + Ø(NDP) vs 10 μM FeCl_3_+ 500 μM CoSO_4_].

To determine the efficacy of iron antagonizing molecule and depolymerase producing phage (KPO1K2) alone or in combination in eradicating the biofilms of *K*. *pneumoniae* B5055 grown in M9 media supplemented with 10 μM FeCl_3_, phage was added at a MOI of 1 to wells containing 10 μM FeCl_3_ and/or 10 μM FeCl_3_ along with 500 μM CoSO_4_. The results presented in Figure 3 show that addition of 500 μM CoSO_4_ or KPO1K2 to the wells containing 10 μM FeCl_3_ resulted in a significant decrease (p < 0.05) of ~2 log for the younger biofilms (1–3 day old) in comparison to control wells supplemented with 10 μM FeCl_3_ alone. There was no significant reduction (p > 0.05) in bacterial count of the older biofilms (4–7 day old). Addition of 500 μM CoSO_4_ as well as phage in 10 μM FeCl_3_ supplemented wells resulted in complete eradication of 1^st^ and 2^nd^ day biofilms (p < 0.005). A significant reduction (p < 0.05) of ~2 log was observed in 3^rd^ and 4^th^ day biofilms in comparison to biofilms treated with cobalt or phage individually. 5^th^ day onwards a consistent reduction of ~0.5-1 log_10_ CFU/ml was observed in wells with cobalt and/or phage alone as well as in combination when compared with control biofilms containing 10 μM FeCl_3_ supplemented media. These results indicated that CoSO_4_ and phage when added in combination although resulted in complete eradication of younger biofilm but had a very little inhibitory effect on the older biofilms of *K*. *pneumoniae* B5055 [Figure 3].

To determine the efficacy of non-depolymerase producing phage (NDP) in eradicating the biofilms of *K*. *pneumoniae* B5055, it was added alone and along with 500 μM of CoSO_4_ in minimal media supplemented with 10 μM FeCl_3_. Results indicated that treatment with phage alone resulted in a reduction of ~1 log on younger biofilms as shown in Figure 3. However, the phage was totally ineffective for older biofilms (4^th^ day onwards). On the other hand, treatment with 500 μM cobalt alone could significantly inhibit biofilm formation till 4^th^ day (p < 0.05) but later on became ineffective, for older biofilms. Treatment with non-depolymerase producing phage and chelator in combination had no additive effect on biofilm eradication in comparison to biofilms treated with depolymerase producing phage and CoSO_4_ in combination (Figure 3).

### Growth and treatment of *Klebsiella pneumoniae* B5055 biofilm formed on coverslip

**Figure 4:**
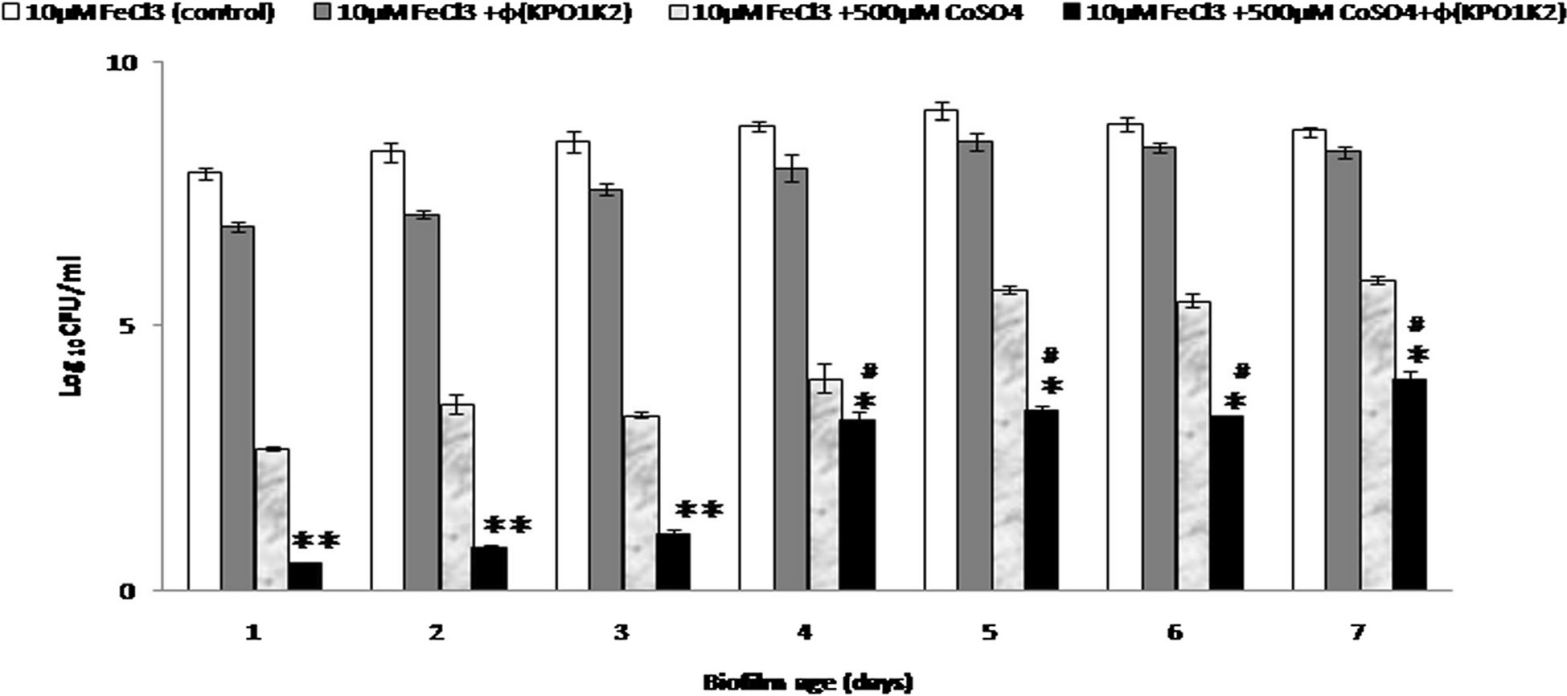
**Kinetics of biofilm formation (on cover slips) by *K. pneumoniae *B5055 grown in minimal media (M9) supplemented with 10** **μM FeCl**_**3 **_**and treated with 500** **μM cobalt salt (CoSO**_**4**_**) and bacteriophage (KPO1K2) alone as well as in combination.** **p < 0.005 [(10 μM FeCl_3_ +500 μM CoSO_4_ + Ø(KPO1K2) vs 10 μM FeCl_3_/10 μM FeCl_3_+ 500 μM CoSO_4_/10 μM FeCl_3_+ Ø(KPO1K2)], *p < 0.05 [(10 μM FeCl_3_ +500 μM CoSO_4_ + Ø(KPO1K2) vs 10 μM FeCl_3_+ 500 μM CoSO_4_], ^#^p < 0.005 [(10 μM FeCl_3_ +500 μM CoSO_4_ + Ø(KPO1K2) vs 10 μM FeCl_3_/10 μM FeCl_3_+ Ø(KPO1K2)].

Besides studies carried out in microtiter wells, biofilm of *K*. *pneumoniae* B5055 was established in iron and iron + cobalt supplemented minimal media on coverslips and were exposed to phage KPO1K2 (MOI = 1) for 24 hours. A higher surface area i.e. 324 mm^2^ of the cover slip allowed enhanced biofilm formation by approximately ~1 log on the cover slip in comparison to the microtiter plate (surface area = 32 mm^2^). Estimation of bacterial numbers in untreated biofilms at the air–liquid interface showed an increase, with a peak on 5^th^ day (9.09 ± 0.15 Log_10_ CFU/ml) of incubation, after which the biofilm bacterial counts decreased progressively (Figure 4). In biofilm treated with both phage and cobalt salt a mean log reduction of ~5 and ~ 2 logs was observed in comparison to the groups treated with phage or iron antagonizing molecule alone. The growth and treatment efficacy of biofilm formed at the air–liquid interface was ~1-2 logs better in comparison to biofilms grown in microtiter plates therefore for further experiments biofilm were grown on glass coverslips at the air–liquid interface. On 3^rd^ and 7^th^ day, the bacterial viability in the treated/untreated biofilms was assessed by fluorescent microscopy.

### Assessment of fluorescent stained biofilms on coverslip

**Figure 5:**
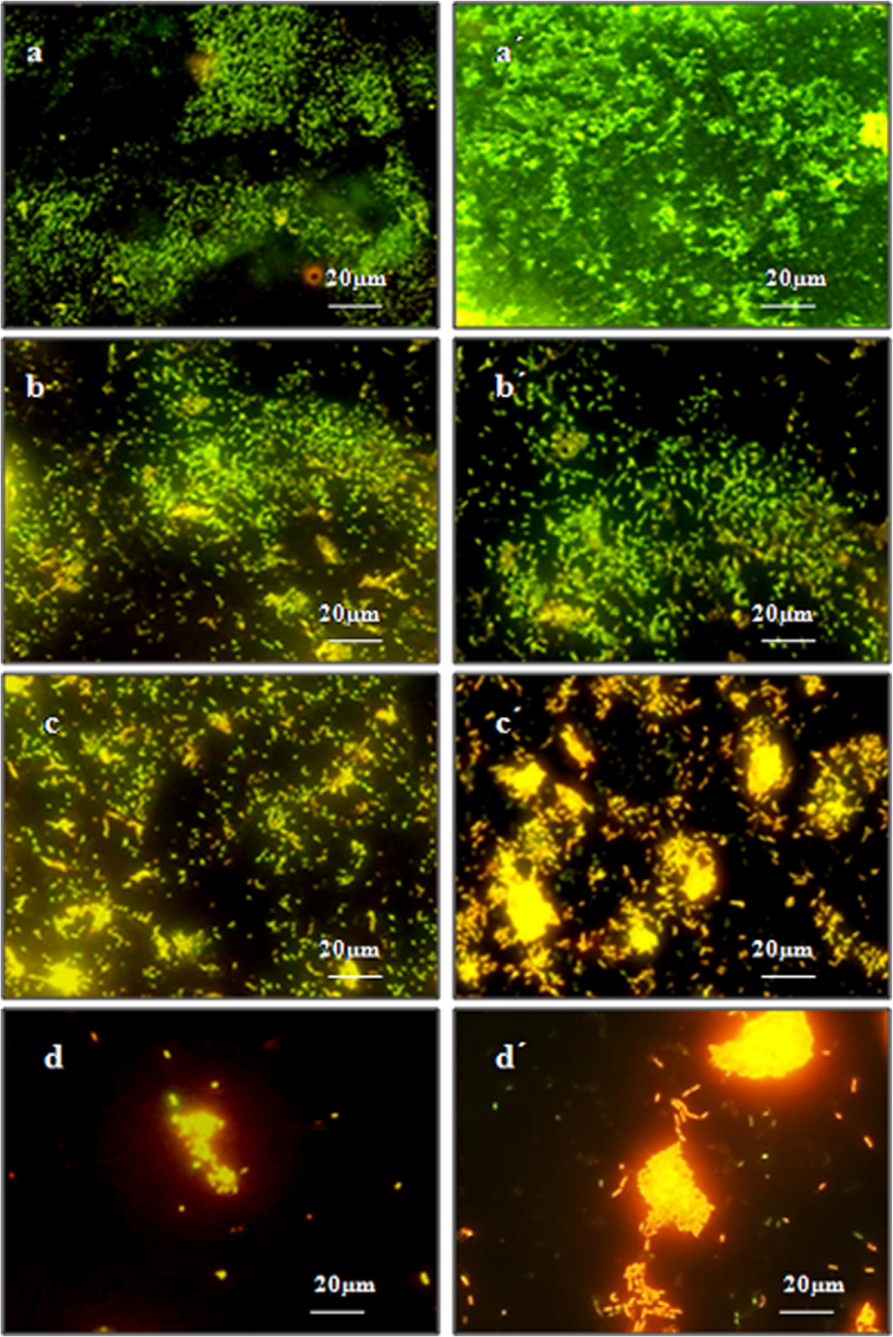
***K. pneumoniae *B5055 biofilm developed on coverslip (a/a´) 3/7 day biofilm grown in 10** **μM FeCl**_**3 **_**supplemented media (b/b´) 3/7 day biofilm grown in 10** **μM FeCl**_**3**_** + 500 μM cobalt salt supplemented media (c/c´) 3/7 day biofilm grown in 10** **μM FeCl**_**3 **_**supplemented media followed by treatment with phage KPO1K2 (d/d´) 3/7 day biofilm grown in 10** **μM FeCl**_**3**_ **+** **500** **μM cobalt salt supplemented media followed by treatment with phage KPO1K2.**

The LIVE/DEAD *BacLight* Bacterial Viability Kit has a mixture of SYTO® 9 green-flourescence nucleic acid stain (for intact live bacteria) and propidium iodide red flourescence nucleic acid stain (for membrane damaged or killed bacteria). Two types of cells were seen, green cells represented the intact or viable cells, red stained cells represented damaged or killed bacterial cells after treatment while yellow regions showed the presence of both red and green coloured cells. As shown in [Figure 5(a)] a 3^rd^ day biofilm consisting of sparsely populated green coloured rods formed in the iron supplemented media in comparison to 7^th^ day old thicker and densely populated green coloured biofilm [Figure 5(a´)]. On the other hand, biofilm grown in additional cobalt supplemented media showed a lesser confluent growth of green colored cells along with some yellow and red cells on 3^rd^ day [Figure 5(b)] as well as on 7^th^ day [Figure 5(b´)] in comparison to biofilms grown in iron supplemented media. 3^rd^ day biofilm grown in iron supplemented media treated with phage alone consisted of sparsely populated green cells with many red coloured rods [Figure 5(c)] in comparison to 7^th^ day biofilm grown in iron supplemented media and treated with phage alone where although a denser biofilm was formed but compared to 3^rd^ day biofilm, it consisted of maximum number of red colored cells in comparison to the green cells [Figure 5(c´)]. When biofilms grown in iron supplemented media were treated with cobalt as well as phage in combination, a negligible amount of biofilm formation consisting of mostly of red and yellow regions was seen on day 3 [Figure 5(d)] as well as on day 7 [Figure 5(d´)] when compared with 3^rd^ and 7^th^ day biofilms treated with cobalt as shown in [Figure 5(b) and Figure 5(b´)] respectively or phage alone [Figure 5(c) and Figure 5(c´)].

## Discussion

Biofilms are recalcitrant to antibiotics as their higher concentrations are needed to eradicate bacterial cells in this mode of growth. Attempts have been made in the past to evolve alternate strategies to destroy biofilms. Since bacteria, both in planktonic and biofilm mode require iron for their growth [14] hence, iron chelating agents have been reported to inhibit biofilm growth. Hancock et al. [15] have reported that since Zn (II) and Co (II) have a higher than iron affinity for the master controller protein of iron uptake i.e. ‘Fur’ thus they reduce biofilm formation by infectious *E*. *coli*. In this study, a significant reduction (p < 0.05) was observed in the growth of younger biofilms (1–3 day old) when 500 μM CoSO_4_ and 10 μM FeCl_3_ supplemented media was used. This might be because of the elevated levels of metals which could interfere with normal iron regulation by shutdown of Fur-controlled iron uptake systems like enterobactin, ferric dicitrate, aerobactin as well as additional downstream effects on putative adhesion factors involved in biofilm establishment thereby resulting in deleterious effect on biofilm formation [2,22] as well as pathogenicity of the organism.

No previous reports are available involving the use of phage and iron antagonizing molecules in combination on biofilm kinetics. Thus, we studied the efficacy of depolymerase producing phage (KPO1K2) in eradicating the biofilms of *K*. *pneumoniae* B5055 grown in minimal media supplemented with 500 μM CoSO_4_ and iron. A complete eradication of the younger biofilms (upto 2 day old) given combination treatment was observed. This was possibly due to the degradation of exopolysaccharide matrix encompassing the biofilm structure by the phage encoded depolymerase [7,17] which facilitated the process of bacterial growth inhibition by phage as well as CoSO_4_. These results suggests that prior addition of CoSO_4_ and later treatment with depolymerase producing phage is quite effective in degrading biofilms. Furthermore, when biofilms of different ages were treated with non-depolymerase producing phage (NDP) alone as well as in combination with CoSO_4_, a less reduction in overall bacterial load was observed in comparison to biofilms treated with depolymerase producing phage and CoSO_4_ together. These findings suggest that this might be due to the degradation of exopolysaccharide matrix of biofilm by depolymerase enzyme that facilitated the diffusion of cobalt ions.

Qualitative analysis of viability of biofilms treated with phage in the presence and absence of cobalt ions was further done by staining with LIVE/DEAD BacLight Bacterial Viability Kit. Appearance of maximum number of dead cells and formation of thin biofilms indicated the effectiveness of the combined treatment with CoSO_4_ and bacteriophage. Previous works by O’May et al. [14] and Reid et al. [23] have also reported inhibition in *P*. *aeruginosa* biofilm formation by iron chelator and tobramycin when observed by staining with BacLight Bacterial Viability staining kit.

## Conclusion

Since, a rise in antimicrobial resistance has made the chase for development of newer antimicrobials especially against biofilm related infections necessary and also because of the various advantages bacteriophages offer over antibiotic treatment they can be used alone as well as in combination with the other therapies such as iron chelators/antagonizing molecules. This strategy although needs further exploration particularly for *in vivo* applications, but can be exploited for coating of devices with iron chelators to reduce biofilm formation and subsequent treatment of established biofilms with phages as adjuncts to the already available antibiotics.

## Competing interests

The authors declare that they have no competing interests.

## Authors’ contributions

SC, SB: Conceived and designed the experiments; DN: Performed the experiments; SC, SB: Analyzed the data; SC, SB: Wrote the paper. All authors read and approved the final manuscript.
